# Cost-Efficiency Analysis of the Improved Web-Based Planning, Budgeting, and Reporting System (PlanRep) in Tanzania

**DOI:** 10.3389/frhs.2021.787894

**Published:** 2022-01-18

**Authors:** George M. Ruhago, Ntuli A. Kapologwe, Frida N. Ngalesoni, James T. Kengia, Stephen M. Kibusi, Albino Kalolo, Erick J. Kitali, James D. Mtatifikolo, Sutte R. Masuha, Amani Kikula, Gemini Mtei

**Affiliations:** ^1^Department of Development Studies, School of Public Health and Social Sciences, Muhimbili University of Health and Allied Sciences, Dar es Salaam, Tanzania; ^2^President's Office Regional Administration and Local Government (PORALG), Dodoma, Tanzania; ^3^Amref Health Africa Tanzania, Dar es Salaam, Tanzania; ^4^Department of Public Health, University of Dodoma, Dodoma, Tanzania; ^5^Department of Public Health, St. Francis University College of Health and Allied Sciences, Ifakara, Tanzania; ^6^Department of Obstetrics and Gynecologist, School of Medicine, Muhimbili University of Health and Allied Sciences, Dar es Salaam, Tanzania; ^7^Abt Associates, Tanzania Public Sector Systems Strengthening Plus (PS3+) Project, Dar es Salaam, Tanzania

**Keywords:** web-based planning and reporting system, cost efficiency, utilization, timesaving, sustainability

## Abstract

**Background:**

Information systems offer unlimited potential for innovation and digitalization of management functions to facilitate citizen participation and improve accountability, transparency, and efficiency in government operations and service delivery. In line with this, for more than one decade, Tanzania implemented an integrated planning, budgeting, and reporting system (PlanRep) that was used to prepare plans and budgets at the local government authorities (LGAs) using a desktop application. In 2017, PlanRep was upgraded to a Web-based system to address several challenges, including poor coordination and high cost involved in the preparation of plans and budgets. However, operational evidence regarding the cost-efficiencies and benefits of shifting to Web-based PlanRep has not been explored. This study aims to address this gap by assessing efficiency gains (in terms of cost and time) of shifting to a Web-based PlanRep system as a tool for the preparation of LGA plans and budgets.

**Methods:**

The study applied a retrospective before-and-after study design whereby quantitative data was used to assess the amount of time and the cost incurred by LGAs when preparing their budget 1 year before the introduction of PlanRep and 1 year after. Parallelly, qualitative data were collected through key informant interviews with selected LGA officials, Regional Secretariats (RSs), President's Office Regional Administration and Local Government (PORALG), and system end-users such as heads of health facilities and schools (primary and secondary). Secondary data was analyzed by comparing time and cost used before and after Web-based PlanRep, while thematic analysis was employed for qualitative data.

**Results:**

The analysis showed a 53% reduction (from USD 3.8 million in 2017/18 to USD 1.8 million in 2018/19) in the total costs LGAs incurred during planning and budgeting after introducing the Web-based PlanRep. The main efficiency gain was related to per diem costs. The analysis also showed significant time saving from an average of 87 days in 2017/18 to only 8 days in 2018/19. PlanRep system end-users also acknowledged that the introduction of Web-based PlanRep has significantly saved their time and costs in preparation of LGA plans and budget.

**Conclusion:**

The introduction of the Web-based planning, budgeting, and reporting systems has resulted in tremendous cost reduction, time savings, transparency, accountability, and workload reduction. The findings offer operational evidence to guide the implementation and scale up of similar systems in countries that share equivalent circumstances like Tanzania.

## Background

The United Nations (UN) calls for utilization of science and technology for achieving sustainable development (SDG) (1). This pertains to transforming evidence generation, planning, decision-making process, new solutions, and ideas through innovation and digitalization. The government of Tanzania (GoT) is making efforts toward that vision and has recently undergone a period of significant reforms. Among these reforms is the implementation of information systems that focus on increasing participation, accountability, transparency, and efficiency in public sector service delivery. The transformation of information systems entails the introduction of a Web-based planning, budgeting, and reporting system (PlanRep) at local government authorities (LGAs), health facilities, and schools in 2017/18.

PlanRep was initially introduced over a decade ago to assist LGAs in planning, budgeting, monitoring actual implementation, and reporting results. It incorporates national sector strategies and enables LGAs to define their Medium Term Expenditure Framework (MTEF) in relation to their strategic plans, objectives, targets, and activities (2–5). PlanRep was a standalone system with different versions used in different public sectors and each user accessing the system locally on his/her workstation. Users often had different versions of PlanRep, which resulted in producing data in different formats and inconsistent database structure that compromised data integrity. PlanRep did not include service providers or their outputs in plans and budgets, thus limiting provider autonomy including their input to plans and budgets. In addition, the system could not transfer information across government levels, thus making the development of national plans and budgets a difficult and lengthy manual process that was tedious and costly. The costs included travels, printing multiple copies, human resources, and time among others. These issues interfered with the uploading of budgets into the Ministry of Finance and Planning (MoFP) Statistical Budget Analysis Software (SBAS) and the Integrated Financial Management Information System (IFMIS)—MUSE (Mfumo wa Malipo Serikalini). As a result, LGAs must manually upload their annual budgets into SBAS and IFMIS-MUSE, a process that takes months and is riddled with human errors (6).

The improved Web-based PlanRep decentralized to health facilities and schools and consolidated at the LGA assists in planning, budgeting, projecting revenue, and tracking funds received; in actual implementation, it is used for financial (Council Financial Reports—CFR) and non-financial reporting (Council Development Reports—CDR). PlanRep acts as the main information source for integration of community plans and the LGA strategic plan for a specific financial year (7). PlanRep contains tools required for preparing the Comprehensive Council Health Plans (CCHPs) and Facility Health Plans as per Ministry of Health, Community Development, Gender, Elderly and Children (MoHCDGEC) guideline (8). These are the operational tools for the health sector strategic plans at the grassroots level.

The PlanRep system is integrated with Facility Financial Accounting and Reporting System—FFARS—elaborated elsewhere, the government of Tanzania payment IFMIS known as MUSE, Local Government Revenue Collection Information System (LGRCIS), and District Health Information System (DHIS) (7).

## Planning and Budgeting

PlanRep operates on a Web-based platform through a virtual private network (VPN) and Internet with encryption to connect users to the central server. The system enables the President's Office Regional Administration and Local Government (PORALG) and different stakeholders to interact on real-time or periodic planning and budgeting. PlanRep enables the PORALG and LGAs to enter budget ceilings, a process of assigning a given funding source between different departments/units, to determine the total expected resource envelope for the LGA, which it should plan and budget, and to capture the composition of the LGA resource envelope from central government sources.

### Budget Ceiling Allocation and Entering

The Council Planning Officers (DPLO) in collaboration with council management team (CMT) are charged with allocating budget ceilings at the start of the planning and budgeting process. Budget ceilings are financial estimates of expected LGA income from the various sources, which are required in order to determine the total expected own source envelope and central government sources within which it should plan and budget. The DPLO is charged with entering ceilings at the start of the planning process and will amend these during the planning process. After budget approval by the parliament, ceilings cannot be changed. Budget reallocation may be done if necessary during the mid-year review of the budget implementation as per Budget Act No. 11 of 2015 and its regulations of 2015.

### Activity Costing

Costing is required in order to ensure that activities can be carried out within the LGA budget, i.e., with the funds available in the relevant planning period, based on the budget ceiling. The cost of an activity depends on the quantity and price of the different inputs required to carry out that activity. Most activities will require two or more inputs. Inputs may be financial, human, or material resources. Institutions are required to ensure that only those inputs relevant to an activity are considered in order to deliver the activity. Inputs should be entered using actual values and proper units of measurements. Budget officers in cost centers and facilities are responsible for entering costs of activities in the system.

### Scrutinization and Budget Submission

Scrutinization is required to check whether the whole plan is according to the National Budget Guideline and other national strategic instruments, the activities are appropriately costed, ceilings have been adhered to, and proper inputs provided. Scrutinization starts after the budget officers responsible for cost centers have completed the activity costing. Firstly, budget officers in cost centers are responsible for submitting their plan and budget to Head of Department (HODs) in the system. HOD undertakes the assessment and scrutinization. Then, the DPLO scrutinizes all department budgets and submits the approved plan and budget to RS. During scrutinization, the DPLO may forward or return the specific budget in case further corrections are needed from a department.

Secondly, the RS is responsible for assessing and scrutinizing the submitted budget from LGAs. After the formal submission of plan and budget files from the LGAs, the RS receives the files in the PlanRep system and informs sections and units to begin the assessment and scrutinization process. Heads of sections and units will scrutinize the plans and budgets from councils. At this stage, the budget may be forwarded to the next level or returned back for corrections.

Thirdly, at the national level, the PORALG is responsible for assessment and scrutinization of the submitted regional plans and budgets. After formal submission of plan and budget files from the RS, the Director, Policy, and Planning (DPP) at the PORALG receives the files in the PlanRep system and informs responsible sections and units to begin the assessment and scrutinization process. The approved budget is then submitted to the Ministry of Finance and Planning before submitting to the parliament for approval. Also, the system provides a feature for MoFP users to perform scrutinization of the budget using approved criteria in the system.

This study was set to assess the cost-efficiency of the PlanRep Web-based system in terms of costs and time saved as well as the perception and experiences of the end-users of the system in Tanzania's LGAs.

## Methods

### Study Area and Design

Tanzania is a lower middle-income country located in East Africa. With a population of 61,627,284, Tanzania has had a steady economic growth ranging from 5 to 7% prior to COVID-19 but fell to 2.1% in 2020 from 6.8% in 2019 (9). The study area covered 185 LGAs where the PlanRep system is used for planning, budgeting, reporting, and tracking funds through activity implementation.

This study employed a parallel mixed method study design. The quantitative strand was used to assess the cost-efficiency of the Web-based PlanRep system, while the qualitative strand was used to assess the utilization and sustainability of the new system. The two data sources were used to triangulate findings and answer the main objectives.

### Sampling Procedures and Sample Size

The quantitative sample included a survey of all 185 councils in the country. The analysis was done on data from 173 (93.5%) LGAs, which completed the survey comprising of 45 urban and 128 rural councils. The target population included the district council planning officers and council treasurers. The qualitative sample included key informants selected on virtue of their positions and experiences in the planning and budgeting process.

### Data Collection Tools and Procedures

A structured questionnaire was used to collect quantitative data. The questionnaire was sent electronically by email to the DPLO and council treasurers (CT) (see Appendix 2 in the Supplementary Material). The questionnaire involved questions on costs associated with travel and per diems, stationary and printing, hiring meeting venues, and refreshments. DPLO and CT with their teams collaborated in filling the form for each council. Document review was conducted with the aim of documenting all the processes undertaken in preparation of LGA plans and budgets.

A semi-structured interview guide was used to collect data from key informants. A total of 27 key informant interviews (KII) were done virtually by call due to the COVID-19 pandemic with officials from LGAs and RSs (eight interviews); supporting implementing partner, PORALG from Health, Education, Regional Administration, Policy, and Planning; local government and information communication and technology (ICT) departments (eight interviews); and service delivery points (health facility in charges and head of schools) (eight interviews). The interviewees had experience in the planning and budgeting process to build an understanding of the extent of gains that have been achieved from the use of the Web-based PlanRep since its inception. Our interviews focused particularly on exploring end-users' experience on utilizing the new Web-based PlanRep in comparison with the old manual-based, stand-alone system. The interview explored questions around process duration, ease of operation, user-friendliness, communication, perceived cost, time saving, the overall benefits of the systems, and challenges in implementing the upgraded Web-based system. Pretesting of the tools had happened before actual data collection.

### Data Management and Analysis

The key variables of interest in this study included the four typical stages of the budget development and review process in the government, namely, (i) budget preparation at the LGA level (including the lower level facilities); (ii) budget assessment and scrutinization at the regional secretariat level; (iii) budget assessment and scrutinization at the PORALG level; and (iii) budget scrutinization and review at the Ministry of Finance and Planning level. Quantitative data was entered into Microsoft Excel, sequentially cleaned, and analyzed. Cost-efficiency was determined by disaggregating the cost and time used before and after the introduction of Web-based PlanRep. The cost differences used for planning and budgeting at the council level were determined to get the cost saved. The time saved was assessed through analysis of the time it took from the planning stage to submission of the proposed budget to the parliament; the time was compared before and after the introduction of the Web-based PlanRep system.

Qualitative data analysis was done by thematic analysis as described by Braun and Clarke (10). Data was transcribed, then initial familiarization was done through reading and re-reading the texts, and initial codes were developed, from which themes and sub themes were developed.

## Results

### Costs Incurred During Planning and Budgeting Cycle Before and After Web-Based PlanRep

**Figure 1:**
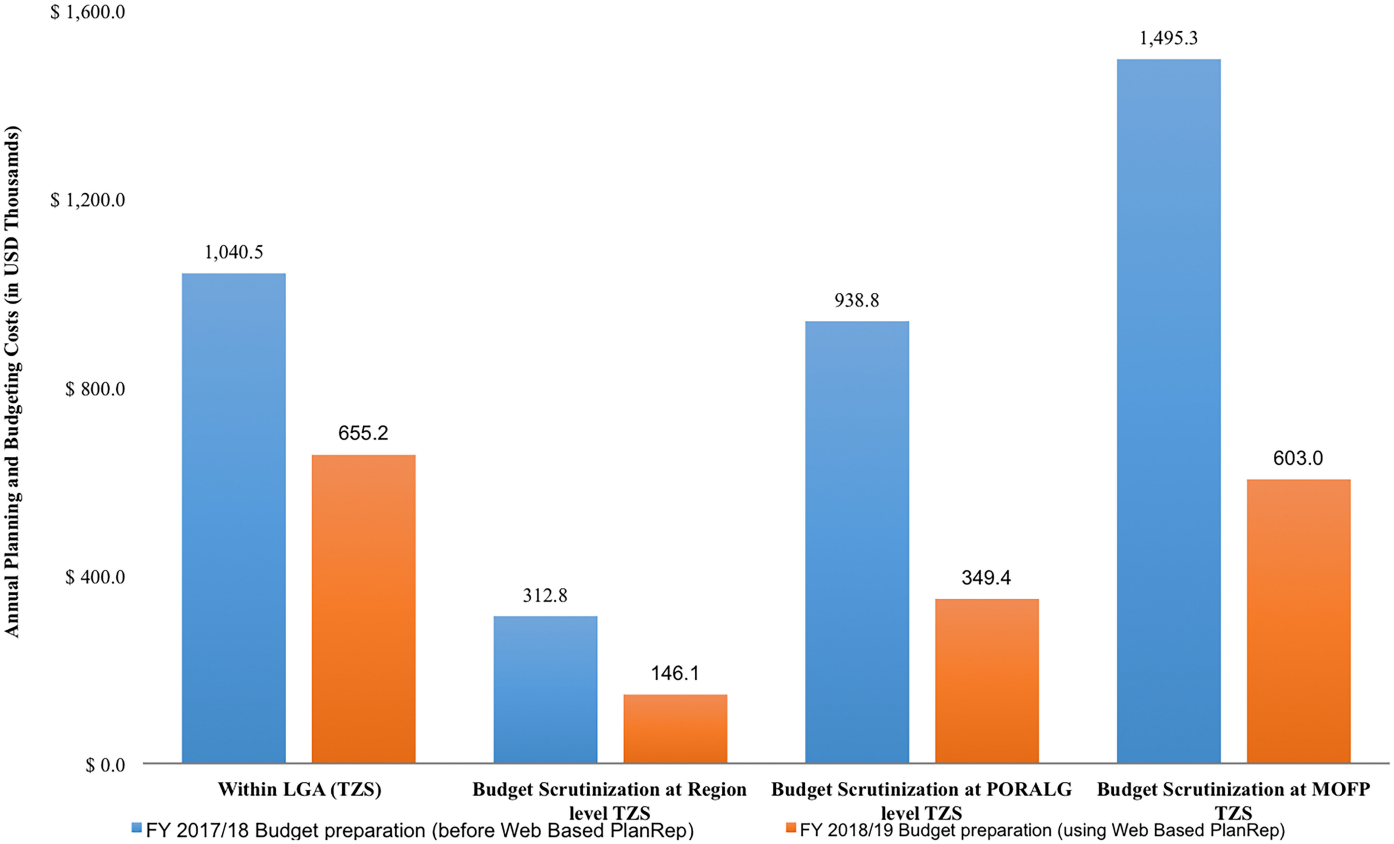
Costs for planning and budgeting process at different levels.

The Web-based PlanRep championed a fifty three percent (53%) reduction in cost incurred for preparing plans and budgets from US$ 3.8 million during financial year 2017/18 to US$ 1.8 million in 2018/2019. There was a significant reduction in the cost of budget assessment and scrutinization at national level, about fifty nine percent (59%) from US$ 1.5 in financial year 2017/18 to US$ 0.6 (Figure 1).

### Itemized Costs for Planning and Budgeting

**Figure 2:**
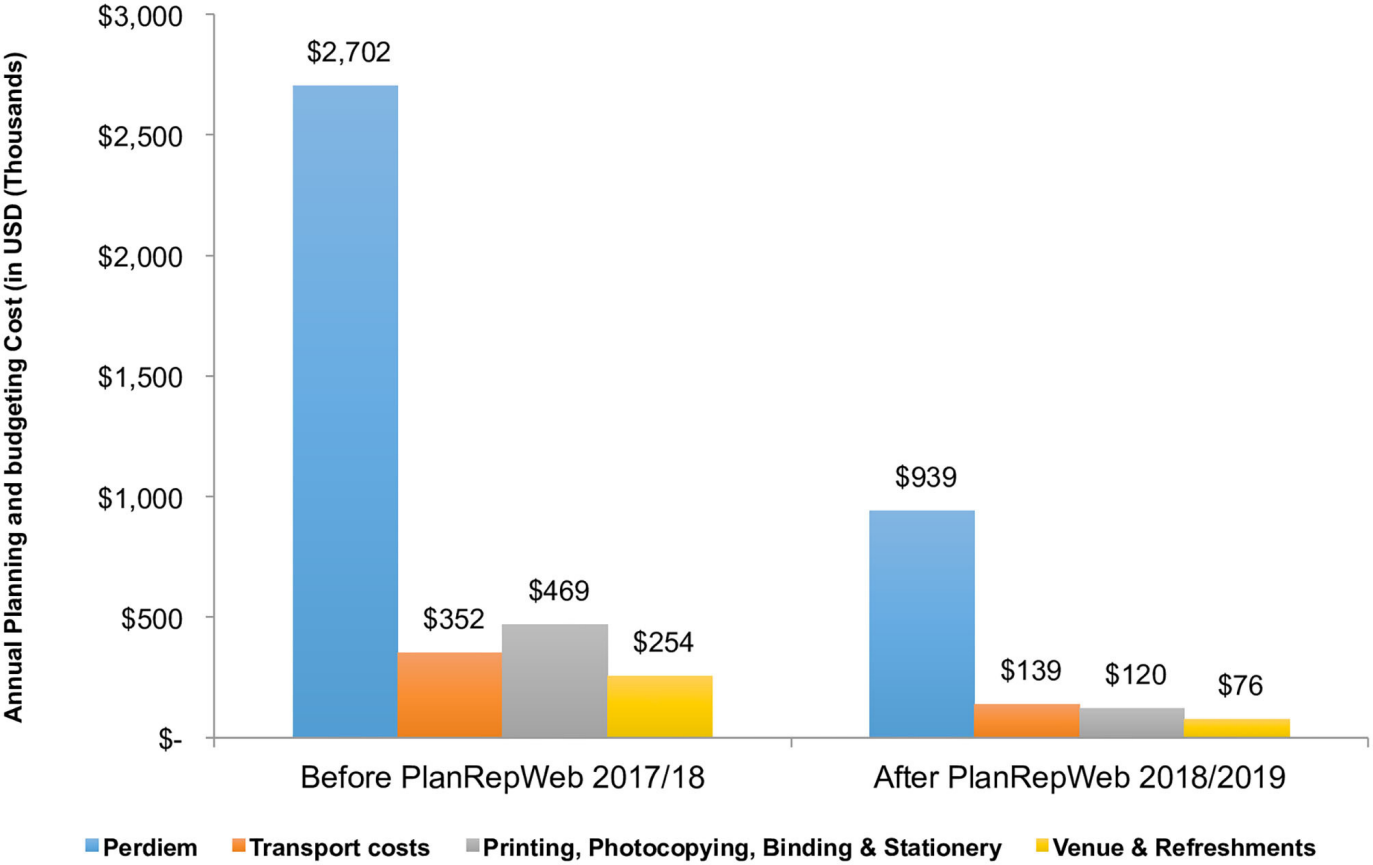
Disaggregated costs for planning and budgeting.

The main cost driver for the planning and budgeting process in both urban and rural LGAs were daily subsistence allowance (DSA) required for personnel traveling from their workstations to centers for planning and budgeting before the new system. After implementing the Web-based PlanRep system, the cost was reduced (by 51%) from US$ 0.6 million in 2017/18 USD to US$ 0.3 million in 2019/20. Stationary costs, the second leading costly component, was reduced (by 61%), from US$ 0.1 million USD in 2017 to US$ 0.05 million in 2018/2019 (Figure 2).

### Time Saved

The analysis of time used for the planning and budgeting process when LGAs used the stand-alone PlanRep tool and the new redesigned Web-based PlanRep demonstrates significant time savings from an average of 87 days to only 8 days, with the most saved time observed during the budget assessment and scrutinization at RS, PORALG, and MoFP, which is now done electronically (Table 1).

**Table 1:** Time spent by LGAs in planning and budgeting before and after web based PlanRep.

	**Step/activity**	**Duration in days (before)**	**Duration in days (after)**	**Responsible (before)**	**Responsible (after)**
1	CPLO compiles and enters a draft LGA plan and budget	30	N/A	CPLO and his/her team + consultation	N/A
2	Department budget officers enter plans and budgets into PlanRep	N/A	2		HoDs and budget officers (100+ roughly) + for SPs (also responsible to enter budget in the PlanRep)
3	RS planning and coordination section scrutinizes budgets and submits to PORALG	10	2	RS budget offices from planning and coordination section	RS budget offices from planning and coordination section
4	PORALG and MoFP scrutinize budgets	30	2	PORALG–DPP + DRA and DLG + sectors	PORALG–DPP + DRA and DLG + sectors
5	LGAs accommodate changes and new ceilings	14	1	CPLOs	AAS planning, CPLOs, and LGA budget officers
6	RS and LGAs accommodate all changes suggested by administration and local government parliamentary committee and submit to MoFP	3	1	RC; RAS; RS budget officers	RC; RAS; RS budget officers and CPLOs
Total days	87	8		

*N/A, Not Applicable*.

KII respondents from PORALG shared the same opinion regarding the time saving saying, for example, that they have been able to significantly reduce both actual time used in budgeting as well as time used for travel related to the budget preparation process. The respondents reported that, in the past, each district council sent a team with about five or more staff for the budget preparations or scrutinization at the PORALG. This is no longer necessary. They can do all the planning while at their workplace and similarly receive and respond to inquiries and feedback from PORALG.

“*The budget cycle is the same. 4–6 Months beginning from October to June, the planning cycle and process remains the same, this begins from Community level, Primary health facility, DMOS, RAS, PORALG. But now they can continue with other work, and preparation of the budget takes much shorter time.”*

“*When we were using the PlanRep desktop application, we had to have all the LGAs coming to Dar es Salaam (DSM) and/or Dodoma during the budget preparation process. Now, however, the budget assessment, scrutinization and submissions are done online.”*

### Utilization of the New System

All stakeholders responsible with GoT systems development, maintenance, and use, e.g., the MoFP and MoHCDGEC as well as e-government authority, were involved to ensure ownership and use. Following the introduction of the Web-based PlanRep, it has enabled the utilization of a standard planning and budgeting template across the facilities, LGAs, RAS, and PORALG;

“*Formerly with a standalone system in 185 LGAs, there was a problem of having different versions of budgets hence most of the time we will have different budgets. The files are different at each level LGAs, RAS, PORALG now we have* (11) *same system, same plan at all levels.”*

The interoperability of the PlanRep system with other available systems such as accounting and reporting systems such as Epicor and FFARS is another significant benefit as it has facilitated automatic transfer of data between the systems. This has reduced the staff burden and time involved in transferring data between systems used at LGAs and eased the retrieval of information as well as the quality and consistency of the information coming from LGAs. Respondents from PORALG stated that

“*Now it has been integrated with other system such as* Epico*r and FFARS via Muunango Gateway (PORALG Interoperability Layer), now PORALG can have real time data within the shortest time possible from all health facilities (5579 Facilities across the country, including DH, HC and Dispensaries).”*

### Sustainability

Proactive engagement of stakeholders at the design stage, which took about 9 months, of the new systems and the desired improvements in the existing systems has been highlighted as key in ensuring that these systems, though supported by implementing partners, are driven by the government to ensure ownership, utilization, and sustainability in the future.

“*Development of new systems or upgrades/redesign of existing systems started with joint discussion between PS3 and PORALG to identify requirements for the systems improvements.”*

A respondent from PORALG recited similar sentiments:

“*From the beginning we started working on common ground, jointly planned, designed, implemented and followed up the execution phase*.”

This in turn built the ownership of the system within the government. As different respondents put it:

“*…the systems are government owned 100 percent.”*

“*There is no turning back to the paper-based system. The government will continue with the systems even after the project ends.”*

Another respondent from PORALG stated,

“*Sustainability is not a big problem. The good thing is that the PS3 project supported government structure from the national to the lower level. So, this is good, district, regional medical officers are aware of these systems and everything is integrated within the government system.”*

At the national level, the MOFP is considering how to make use of PlanRep and make it interoperable with the systems that are used at the central government level for budget planning and reporting. Currently PlanRep is used by LGAs and has been further customized in 2020 to be used by all institutions under the Treasury Registrar. Expanding the use of the systems across the government safeguards sustainability, as respondents noted.

“*…MOFP has to consider using PlanRep to be used as a national planning and budgeting system by linking it with the central government planning budgeting tool, the Central Budget Management System (CBMS) and extending the use of the PlanRep to other ministries.”*

The implementation environment of the systems can facilitate or impede their utilization and sustainability. Respondents raised a number of issues as bottlenecks to the effective utilization of the systems. These included shortage of ICT equipment such as computers and printers, poor internet connectivity including infrastructure such as local area networks (LANs) and servers at the lower levels, and access to electricity and instability of supply in areas where it exists. One respondent at the regional level narrated on ICT equipment,

“*Some rural Councils do not have capacity to buy computers for facilities.”*

Another respondent at the LGA level commented regarding inadequate supply of electricity and Internet connectivity,

“*Biggest challenge is electricity and internet connectivity…you might want to generate a report and the system is not working.”*

## Discussion

This study aimed at assessing the cost-efficiency, utilization, and sustainability of the redesigned Web-based PlanRep system. Our findings revealed a significant resource saving, improved budgeting and planning processes, usability, and likelihood of sustainability after the transformation of the planning and budgeting system.

The transformation not only replaced paper- and desktop-based planning but also involved redesigning the internal planning PlanRep processes that has resulted in an increased efficiency and improved planning process and organizational performance. A study in Italy indicated similar levels of resource savings of about 42–60% after digitizing the organization activities (12).

The extension of the PlanRep system to health facilities through the inclusion of service provider codes with all features complying with the CCHP requirement has enabled them to develop their own plan and budgets. The system interoperability capability has mostly benefited the health sector in the implementation of the DHFF and FFARS—see *References* for more details—that are interoperable with the Web-based PlanRep. This has enhanced the health sector in allocating and tracking resources to the lowest level of health services delivery (13). This is crucial to ensure real-time scrutinization is needed to avoid funding expensive interventions, when cheaper, equally effective options are available, since this could lead to less benefit to the population. In such contexts, it is therefore important that technology informs planning and budgeting processes in order to ensure the effective use of limited resources and re-direction of resources to the grassroots where they are mostly required (14).

We observed that engaging end-users from the onset allowed rapid adoption, deployment, uptake, and use of the redesigned Web-based PlanRep. As a consequence, currently, the system is used by all LGAs, and it is the only permissible means for submission of budget plans. A study in Ghana revealed similar findings that engaging the user point of view in the digital transformation process is an important element to ensure wide use of the systems (15). Similar findings have been reported by Silvius and Schipper (16) that the key to sustainability is consideration and valuing the potential interests of stakeholders. Furthermore, the ISO (17) has provided guidance on the importance of proactive stakeholder engagement as key in ensuring project sustainability is achieved. Stakeholder participation therefore requires “a process of dialogue and ultimately consensus-building of all stakeholders as partners who together define the problems, design possible solutions, collaborate to implement them, and monitor and evaluate the outcome.”

The finding that the system has allowed workers to develop the budgets within their own work locations has implications in permitting critical workers such as health workers and teachers to spend more time attending patients and teaching rather than commuting between their workstation and regional and national levels seeking for approvals of their plans and budgets.

However, the implementation of the new system at the lower level has faced a number of challenges including notable shortage of ICT equipment such as computers and printers that complicates the utilization of the systems and compelling lower-level and rural facilities to utilize internet cafés and stationary shops in accessing the system and printing FFARS vouchers and PlanRep reports. The lack of ICT equipment mostly affects new LGAs and small health facilities with low income. A focused approach to lower-level facilities to enable the use of the systems could enable further efficiency gains.

Other limitations to be considered while interpreting these results are as follows: firstly, this study was done only 2 years after the implementation of the Web-based PlanRep; hence, we might have underestimated the potential impact of the improved Web-based PlanRep. Secondly, our interviews were done virtually due to the COVID-19 pandemic, and this might have limited the potential of further interactions and observations of the systems use. However, its effect on the magnitude and direction of the findings reported is very minimal owing to the fact the nature of the research question does not carry emotion deductions that could be compromised by a virtual interview. Addressing each of these questions will be essential in understanding the full benefits of the improved Web-based PlanRep.

## Conclusions

The redesigned Web-based PlanRep system has significantly improved budgeting, planning, and reporting processes. There are both monetary and non-monetary efficiency gains in terms of time savings and streamlined workflows that both LGAs and national-level PORALG/MOFP experienced. Nonetheless, there is potential for further cost and time savings as the system continues to evolve and information technology (IT) infrastructure is extended to health facilities and schools with limited capabilities in technology and utilization of the application to develop their own plans and budgets.

## Data Availability Statement

The original contributions presented in the study are included in the article/Supplementary Material, further inquiries can be directed to the corresponding author/s.

## Author Contributions

GR collected all the primary cost and quantitative data, conducted the analyses, and wrote the first draft. All authors contributed to the research design, data collection, interpretation of the results, and writing the manuscript.

## Conflict of Interest

The authors declare that the research was conducted in the absence of any commercial or financial relationships that could be construed as a potential conflict of interest.

## Publisher's Note

All claims expressed in this article are solely those of the authors and do not necessarily represent those of their affiliated organizations, or those of the publisher, the editors and the reviewers. Any product that may be evaluated in this article, or claim that may be made by its manufacturer, is not guaranteed or endorsed by the publisher.
