# Supplementary material for: Cost-Efficiency Analysis of the Improved Web-Based Planning, Budgeting, and Reporting System (PlanRep) in Tanzania
Source: Front Health Serv. 2022 Jan 18;1:787894. doi: 10.3389/frhs.2021.787894 (PMC10012613; doi:10.3389/frhs.2021.787894)
Supplement: Supplementary file 1 [file Data_Sheet_1.docx]

**Appendix 1: Details of LGAs involved in the analysis**

| **SN** | **REGION** | **LGA CATEGORY** | **LGAs** |
| --- | --- | --- | --- |
| 1 | DODOMA | Urban | Dodoma CC and Kondoa TC |
|  |  | Rural | Chemba, Kondoa, Mpwapwa, Bahi and Kongwa DCs |
| 2 | MBEYA | Urban | Mbeya CC |
|  |  | Rural | Busokelo; Chunya; Kyela, Mbarali & Rungwe DCs |
| 3 | KAGERA | Urban | Bukoba MC |
|  |  | Rural | Missenyi; Biharamulo; Ngara; Muleba; Karagwe; and Bukoba DCs |
| 4 | MTWARA | Urban | Mtwara Mikindani MC; Nanyamba TC; Nanyumbu TC; Masasi TC & Newala TC |
|  |  | Rural | Masasi; Mtwara & Tandahimba DCs |
| 5 | MWANZA | Urban | Mwanza CC and Ilemela MC |
|  |  | Rural | Kwimba; Ukerewe, Buchosa; Misungwi; Magu and Sengerema DCs |
| 6 | SHINYANGA | Urban | Shinyanga MC and Kahama TC |
|  |  | Rural | Msalala; Shinyanga; Ushetu and Kishapu DCs |
| 7 | IRINGA | Urban | Iringa MC and Mafinga TC |
|  |  | Rural | Iringa; Kilolo; and Mufindi DCs |
| 8 | KIGOMA | Urban | Kigoma Ujiji MC and Kasulu TC |
|  |  | Rural | Kigoma; Uvinza; Kibondo; Kasulu; Kakonko and Buhigwe DCs |
| 9 | NJOMBE | Urban | Makambako TC and Njombe TC |
|  |  | Rural | Wanging'ombe; Makete and Ludewa DCs |
| 10 | LINDI | Urban | Lindi MC |
|  |  | Rural | Lindi; Nachingwea; Liwale; Ruangwa and Kilwa DCs |
| 11 | DAR-ES-SALAAM | Urban | Dar CC; Kinondoni MC; Ilala MC and Ubungo MC |
|  |  | Rural |  |
| 12 | COAST | Urban | Kibaha TC |
|  |  | Rural | Kibaha; Rufiji; Bagamoyo; Chalinze; Kibiti; Mafia and Mkuranga DCs |
| 13 | TABORA | Urban | Tabora MC and Nzega TC |
|  |  | Rural | Urambo; Sikonge; Nzega; Igunga; Kaliua and Uyui DCs |
| 14 | KILIMANJARO | Urban | Moshi MC |
|  |  | Rural | Moshi; Siha; Same; Rombo; Hai and Mwanga DCs |
| 15 | SIMIYU | Urban | Bariadi TC |
|  |  | Rural | Bariadi; Busega; Meatu and Itilima DCs |
| 16 | SONGWE | Urban | Tunduma TC |
|  |  | Rural | Ileje; Mbozi; Momba and Songwe DCs |
| 17 | ARUSHA | Urban | Arusha CC |
|  |  | Rural | Arusha; Karatu; Longido; Monduli and Ngorongoro DCs |
| 18 | MANYARA | Urban | Babati TC |
|  |  | Rural | Hanang; Kiteto; Mbulu; Simanjiro and Babati DCs |
| 19 | GEITA | Urban | Geita TC |
|  |  | Rural | Chato; Geita; Mbogwe; Nyang'hwale and Bukombe DCs |
| 20 | MOROGORO | Urban | Ifakara TC |
|  |  | Rural | Ulanga; Malinyi; Kilosa; Gairo; Mvomero and Morogoro DCs |
| 21 | KATAVI | Urban | Mpanda MC |
|  |  | Rural | Nsimbo; Mpimbwe; Mpanda and Mlele DCs |
| 22 | MARA | Urban | Musoma MC; Bunda TC and Tarime TC |
|  |  | Rural | Tarime; Musoma; Bunda; Butiama; Rorya and Sengerema DCs |
| 23 | RUVUMA | Urban | Songea MC and Mbinga TC |
|  |  | Rural | Madaba; Mbinga; Namtumbo; Songea; and Tunduru DCs |
| 24 | SINGIDA | Urban | Singida MC |
|  |  | Rural | Ikungi; Iramba; Itigi; Manyoni; Mkalama and Singida DCs |
| 25 | TANGA | Urban | Tanga CC; Korogwe TC and Handeni TC |
|  |  | Rural | Pangani; Muheza; Mkinga; Lushoto; Kilindi; Handeni; Bumbuli and Korogwe DCs |
| 26 | MTWARA | Urban | Mtwara; Nanyamba; Nanyumbu; Masasi and Newala TCs |
|  |  | Rural | Masasi; Mtwara; Tandahimba and Newala DCs |

**Appendix 2: Financial Costs for planning and budgeting before and after web based PlanRep urban/rural disaggregated**

| **Table A: FY 2017/18 Budget preparation (before Web Based PlanRep)** | | | | | | | |
| --- | --- | --- | --- | --- | --- | --- | --- |
| **Cost Item** | **LGA Category** | **No. of LGAs** | **Within LGA (USD)** | **Budget Scrutinization at Region level USD** | **Budget Scrutinization at PORALG level USD** | **Budget Scrutinization at MOFP USD** | **TOTAL (USD)** |
| **FY 2017/18 Budget preparation (before Web Based PlanRep)** | | | | | | | |
| Perdiem | Urban | 45 | 166,593 | 29,226 | 148,666 | 329,171 | **673,656** |
|  | Rural | 128 | 545,809 | 174,787 | 518,290 | 799,322 | **2,028,400** |
|  | *All LGAs* | *173* | *712,402* | *204,014* | *666,956* | *1,128,493* | ***2,702,056*** |
| Transport Costs | Urban | 45 | 17,398 | 3,145 | 32,276 | 47,850 | **100,669** |
|  | Rural | 128 | 29,624 | 24,485 | 85,137 | 112,530 | **251,776** |
|  | *All LGAs* | *173* | *47,022* | *27,630* | *117,414* | *160,380* | ***352,445*** |
| Printing, Photocopying, Binding & Stationery | Urban | 45 | 51,608 | 20,139 | 34,384 | 47,293 | **153,423** |
|  | Rural | 128 | 83,491 | 50,549 | 97,370 | 100,073 | **315,432** |
|  | *All LGAs* | *173* | *135,099* | *70,688* | *115,702* | *147,366* | ***468,855*** |
| Venue & Refreshments | Urban | 45 | 40,451 | 1,017 | 3,803 | 14,729 | **60,000** |
|  | Rural | 128 | 105,574 | 9,477 | 34,954 | 44,283 | **194,287** |
|  | All LGAs | *173* | *146,025* | *10,493* | *38,757* | *59,012* | ***254,287*** |
| **GRAND TOTAL - FINANCIAL COSTS** | **All LGAs** | **173** | **1,040,548** | **312,824** | **938,829** | **1,495,251** | **3,777,643** |
| **FY 2018/19 Budget preparation (using Web Based PlanRep)** | | | | | | | |
| Perdiem | Urban | 45 | 131,683 | 16,536 | 72,550 | 131,812 | **352,580** |
|  | Rural | 128 | 350,719 | 81,874 | 173,424 | 323,948 | **938,728** |
|  | *All LGAs* | *173* | *482,402* | *107,173* | *245,974* | *455,760* | ***1,291,308*** |
|  | Urban | 45 | 8,379 | 3,085 | 21,492 | 22,247 | **55,203** |
| Transport Costs | Rural | 128 | 18,291 | 11,283 | 37,845 | 62,309 | **138,491** |
|  | *All LGAs* | *173* | *26,670* | *14,368* | *59,337* | *84,555* | ***184,931*** |
|  | Urban | 45 | 22,202 | 5,565 | 11,856 | 16,567 | **56,190** |
| Printing, Photocopying, Binding & Stationery | Rural | 128 | 46,354 | 15,449 | 24,149 | 34,224 | **120,154** |
|  | *All LGAs* | *173* | *68,556* | *21,015* | *36,005* | *50,791* | ***176,366*** |
|  | Urban | 45 | 20,403 | 66 | 1,610 | 3,159 | **25,238** |
| Venue & Refreshments | Rural | 128 | 57,126 | 3,445 | 6,440 | 8,756 | **75,766** |
|  | All LGAs | *173* | *77,528* | *3,510* | *8,050* | *11,915* | ***101,004*** |
|  |  |  |  |  |  |  |  |
| **GRAND TOTAL - FINANCIAL COSTS** | **All LGAs** | **173** | **655,156** | **146,066** | **349,366** | **603,021** | **1,753,609** |

**Appendix 3: Planning and budgeting process before and after web based PlanRep**

| NO | STEP/ACTIVITY | Duration in Days | Duration in Days | Responsible | Responsible |
| --- | --- | --- | --- | --- | --- |
|  |  | **(Before)** | **(After)** | **(Before)** | **(After)** |
| 1 | RS initiate budget process by writing letter of instructions to RS HODs and LGAs Council Director (CD) | 1 | 1 | RAS through RS Planning & Coordination Section (AAS - PC) | RAS through RS Planning & Coordination Section (AAS - PC) |
| 2 | LGAs initiate budget process by writing letter of instructions and provide indicative planning figures (IPF) to Lower Level of government (VEOs/WEOs) | 1 | 1 | CD through Council Planning Officer (CPLO) | CD through Council Planning Officer (CPLO |
| 3 | Lower Level Governments (LLGs) especially Villages prepare their plans by using O & OD Planning Methodology | 30 | 30 | Village Executive Officers (VEOs); Extension Officers | Village Executive Officers (VEOs); Extension Officers |
| 4 | Service facilities e.g. dispensaries, health centres, schools etc. prepare their plans and submit to Village Council | 7 | 7 | Service Providers | Service Providers |
| 5 | Village Council Meeting convened to discuss Village Plan before submitted to Village Assembly | 2 | 2 | VEO; Village council (25 members); Extension Officers within the village | VEO; Village council (25 members); Extension Officers within the village |
| 6 | Village Plans discussed and Approved by Village Assembly before submitted to Ward Development Committee (WDC) | 1 | 1 | VEO; Village Government (Members are all residents above 18 years in the village) | VEO; Village Government (Members are all residents above 18 years in the village) |
| 7 | Ward Development Committees (WDCs) convened to discuss Village Plans. WDCs must prioritize Village Plans before submitted to Council – CD (DPLO) | 3 | 3 | WEOs; WDC members (depends on the number of villages); Extension Officers within the Ward | WEOs; WDC members (depends on the number of villages); Extension Officers within the Ward |
| 8 | CPLOs submit Ward plans to each respective department for consideration in their departments e.g. construction of classroom projects submitted to the education department etc. | 1 | 1 | CPLO | CPLO |
| 9 | HODs prepare their plans including CCHP under the guidance of CPLO | 10 | 10 | HODs; CPLO; CD | HODs; CPLO; CD |
| 10 | CMT meeting convened to discuss Council Plan for the current year including Ward Plans to be incorporated in the Council Plan | 1 | 1 | CD; HOD (13 departments, 6 sections) | CD; HOD (13 departments, 6 sections) |
| 11(a) | CPLO compiles draft LGA Plan and Budget and enters in the old PlanRep | 30 | N/A | CPLO and his/her team + consultation | N/A |
| 11(b) | Budget officers from each department enters plans and budget into the new PlanRep | N/A | 2 |  | HoDs & Budget Officers (100 + roughly). + for SPs (also responsible to enter budget in the PlanRep) |
| 12 | Stakeholders meeting to discuss LGAs plan and budget for current year (i.e. stakeholders with direct influence at the LGA e.g. businessmen, workers’ unions etc) | 1 | 1 | DAS, CD; HODs and invited stakeholders (or DC chairs) | DAS, CD; HODs and invited stakeholders |
| 13 | Workers Council convened to discuss institutional plan & budget (RS/LGAs) | 1 | 1 | Accounting Officers, HODs and members of workers council | Accounting Officers, HODs and members of workers council |
| 14 | RS convenes meeting with LGAs to discuss Regional priorities | 1 | 1 | RAS; RS HOD; CDs; 3 LGAs Budget Officers | RAS; RS HOD; CDs; 3 LGAs Budget Officers |
| 15 | DC convenes DCC Meeting (special for budget) | 1 | 1 | DC, DAS, CD & HoDs, Other GoT Institution in the District, Stakeholders 30 + invited | DC, DAS, CD & HoDs, Other GoT Institution in the District, Stakeholders 30 + invited |
| 16 | CMT incorporates inputs from Region into council plan and budget proposal. | 1 | 1 | CMT (CPLO) | CMT (CPLO)) |
| 17 | Standing committees meetings except Finance Committee (3 Committees @ 1 day) | 3 | 3 | Respective Standing committees | Respective Standing committees |
| 18 | Finance Committee Meeting to discuss LGA Plan and Budget | 2 | 2 | Finance Committee members | Finance Committee members |
| 19 | Full Council convened to discuss and Approve LGA Plan and Budget | 3 | 3 | Full council members; CD; HODs. Residents are allowed to attend | Full council members; CD; HODs. Residents are allowed to attend |
| 20 | RS Planning & Coordination Section scrutinizes LGAs (based on books submitted) budgets and afterwards submits to PO-RALG | 10 | 2 | RS budget Offices from Planning & Coordination Section | RS budget Offices from Planning & Coordination Section |
| 21 | RS budget committee invited to discuss RS plan & budget | 1 | 1 | RAS, HODs & Sections | RAS, HODs & Sections |
| 22 | RCC convened to discuss and approve Regional budget for current year | 1 | 1 | RCC members (38) and invited members (150) | RCC members (38) and invited members (150) |
| 23 | LGA Plans and Budgets are scrutinized at PORALG | 30 | 2 | PORALG – DPP + DRA, DLG + Sectors | PORALG – DPP + DRA, DLG + Sectors |
| 24 | MoFP organizes Budget Scrutinization process to discuss LGAs budget requests for current year (depending on time table) | 14 | 14 | MoFP: ACB (Regions & LGAs), Accounting Officers of each Vote and their Budget Officers | MoFP: ACB (Regions & LGAs),RAS, CD & CPLO |
| 25 | LGAs accommodate all changes and new ceilings | 14 | 1 | CPLOs | AAS Planning, CPLOs & LGAs Budget Officers |
| 26 | Regional Budget (vote 80) submitted to Administration and Local Government Parliamentary Committee | 9 | 9 | Members of Parliamentary Committee; RC; RAS; CDs & RS budget Officers | Members of Parliamentary Committee; RC; RAS; CDs & RS budget Officers |
| 28 | RS & LGAs accommodate all changes suggested by Administration and Local Government Parliamentary Committee and submit to MoFP | 3 | 1 | RC; RAS; RS budget Officers | RC; RAS; RS budget Officers and CPLOs |
|  |  | ***182*** | ***103*** |  |  |
| 29 | MoFP prepares Government Plan & Budget and submit to Parliamentary Committees | 14 | 14 | Minister MoFP | Minister MoFP |
| 30 | Budget submitted to Parliament for debate and approval | 60 | 60 | Minister MoFP | Minister MoFP |
|  |  | **256** | **177** |  |  |
